# CXCR5 regulates CD8^+^/GZMB^+^ T cell infiltration and apoptotic signaling in breast cancer

**DOI:** 10.1016/j.isci.2026.117874

**Published:** 2026-10-21

**Authors:** Weihong Feng, Jin Yan, Xue Gong, Jiandong Hu, Mengli Jin, Chunhua Ma, Fenge Li

**Affiliations:** 1 Department of Oncology, Tianjin Beichen Hospital, Tianjin 300400, China; 2 Department of Core Laboratory, Tianjin Beichen Hospital, Tianjin 300400, China; 3 Department of Oncology 5, The First Affiliated Hospital of Nankai University, Nankai University, Tianjin 300121, China

**Keywords:** TNBC, CXCR5, paclitaxel, tislelizumab, apoptosis

## Abstract

Emerging evidence associates CXCR5 expression with immunotherapy outcomes; however, the intrinsic role of tumor-derived CXCR5 in modulating chemo-immunotherapy sensitivity in triple-negative breast cancer (TNBC) remains uncharacterized. This study was conducted using murine 4T1 and human MDA-MB-231 TNBC models. Tumor cell CXCR5 expression was manipulated through overexpression or shRNA-mediated silencing. Therapeutic efficacy of paclitaxel (PTX, 10 mg/kg)±Tislelizumab (TIS, 10 mg/kg) was evaluated using *in vivo* tumor models and T cell cytotoxicity assays. We demonstrated that combination therapy effectively suppressed 4T1 tumor growth, extended survival, and enhanced intratumoral CD8^+^/GZMB^+^T cell infiltration coupled with increased cleaved-caspase-3 activation in murine studies. *In vitro* analyses revealed that CXCR5-high MDA-MB-231 cells exhibited heightened sensitivity to T-cell-mediated cytotoxicity in combination with PTX+TIS, corresponding with decreased BCL2 expression and elevated BAX levels. Our findings establish tumor-intrinsic CXCR5 as a pivotal determinant of chemo-immunotherapy efficacy in TNBC, mechanistically linked to enhanced CD8^+^T cell infiltration and apoptotic signaling.

## Highlights

• We found Chemo-immunotherapy enhanced CD8^+^T infiltration and suppressed tumor growth in TNBC
• CXCR5 expression regulates CD8+/GZMB+T cell infiltration and caspase-3 activation in TNBC
• We concluded that tumor-intrinsic CXCR5 is a key regulator in TNBC chemo-immunotherapy
• CXCR5 is a promising target in overcoming primary resistance to chemo-immunotherapy in TNBC

## Graphical abstract

**Figure:**
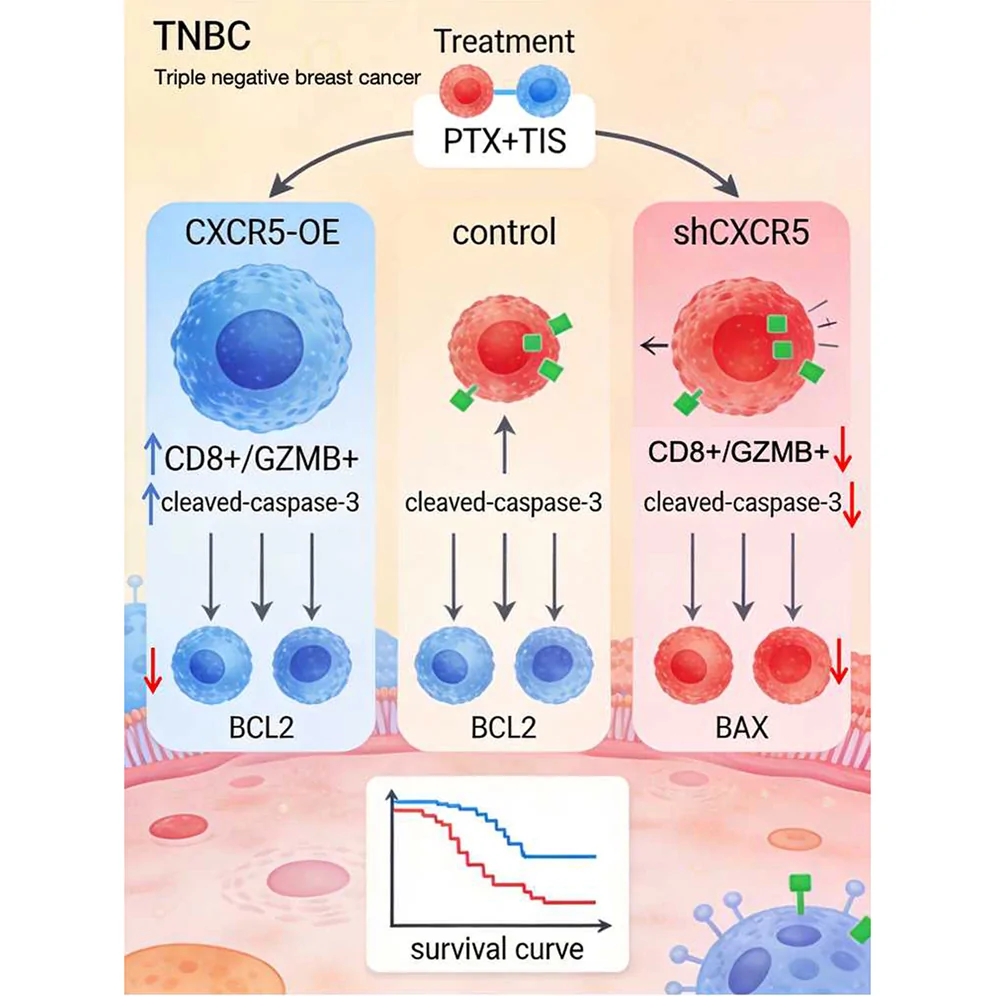

## Teaser

Health sciences; Oncology; Immunology

## Introduction

Triple-negative breast cancer (TNBC) remains the most aggressive subtype of breast cancer, with no approved targeted therapy and a median overall survival (OS) of only 12–18 months in the metastatic setting. Although immune-checkpoint inhibitors (ICIs) such as anti-PD-1/PD-L1 antibodies have transformed the therapeutic landscape of many solid tumors, single-agent response rates in TNBC are <20%.^1^^,^^2^ Consequently, current phase-III trials focus on combination strategies—most commonly PD-1 blockade plus paclitaxel (PTX)-which have improved pathological complete response (pCR) rates from 15% to 35%.^3^^,^^4^^,^^5^ However, the molecular determinants of sensitivity or resistance to this chemo-immunotherapy backbone remain incompletely understood.

Recent single-cell RNA-sequencing (scRNA-seq) studies in TNBC have revealed that therapeutic benefit is tightly linked to the pre-existing immune contexture.^6^^,^^7^^,^^8^ Specifically, the CXC chemokine receptor type 5 (CXCR5) orchestrates critical aspects of immune homeostasis by mediating chemotactic guidance of lymphocytes, sustaining lymphocyte activation dynamics, and governing the formation and maintenance of germinal centers during adaptive immune responses.^9^^,^^10^ CXCR5 also serves as a critical chemokine receptor that governs key aspects of cancer cell biology. This receptor plays a pivotal role in the initiation and progression of multiple human malignancies by modulating fundamental tumor cell behaviors. Moreover, CXCR5 signaling facilitates cancer cell proliferation, enhances invasive capacity, and promotes metastatic dissemination through regulation of growth signaling pathways and cellular motility mechanisms.^11^^,^^12^ CXCL13 is the sole ligand for CXCR5, a G-protein-coupled receptor expressed on Tfc cells, B cells, and a subset of tumor cells.^13^^,^^14^^,^^15^ Mechanistically, CXCL13 drives the formation of tertiary lymphoid structures (TLS), facilitates CXCR5^+^CD8^+^ T cell trafficking, and sustains a stem-like, less-exhausted T cell state that can be reinvigorated by PD-1 inhibition.^16^^,^^17^^,^^18^

The role of CXCR5 signaling in modulating tumor microenvironment dynamics during combination therapy with PD-1 inhibitors and taxanes in TNBC remains undefined, particularly regarding its influence on antitumor immunity and chemotherapeutic responsiveness. Preliminary bulk RNA-seq data generated in our center showed that mRNA of the sole ligand of CXCR5 was the top up-regulated gene in post-treatment biopsies of patients who achieved partial response (PR) to tislelizumab (PD-1 antibody) plus paclitaxel compared with non-partial response. Immunofluorescence confirmed that CXCL13^+^CD8^+^ T cells co-localized with CXCR5-expressing tumor cells within TLS-rich areas, suggesting a functional axis that might amplify both immune-mediated and direct apoptotic signaling.^6^^,^^19^

Building on these observations, the present study was designed to test the hypothesis that CXCR5 is not merely a biomarker but an active effector molecule that enhances the efficacy of PD-1 blockade plus paclitaxel in TNBC. Using human TNBC specimens, syngeneic 4T1 mouse models, and loss- and gain-of-function approaches, we dissected how tumor-intrinsic CXCR5 orchestrates intratumoral T cell infiltration, promotes immunogenic cell death (ICD), and transduces apoptotic signals via the p38 pathway. Elucidating this CXCR5-centered signaling axis may refine patient selection for chemo-immunotherapy and provide a mechanistic rationale for novel combination strategies.

Elucidating this chemokine-receptor-T-cell-infiltration-apoptosis signaling axis may refine patient selection for chemo-immunotherapy and provide a mechanistic rationale for CXCR5-centered combination strategies that could extend survival for women with advanced TNBC.

## Results

### Improved synergistic antitumor efficacy of paclitaxel and Tislelizumab combination therapy compared with chemo-monotherapy

We retrospectively analyzed 20 patients with metastatic TNBC who received either paclitaxel alone (PTX only, *n* = 10) or paclitaxel plus PD-1 inhibitor (PD-1inh + PTX, *n* = 10). Overall survival (OS) was significantly longer in the PD-1inh + PTX group than in the PTX-only group (*p* = 0.003, log rank test; Figure S1A). Progression-free survival (PFS) also favored the combination arm (*p* = 0.001; Figure S1B). These results suggest that the combination of Paclitaxel and Tislelizumab improves survival in patients with triple-negative breast cancer (TNBC).

To further elucidate the role of PD-1 inhibitors in enhancing anti-tumor efficacy against TNBC, we developed a murine model combining PD-1 inhibition with chemotherapy. As shown in Figure 1A, 4T1 triple-negative breast cancer cells were implanted subcutaneously into female C57BL/6N mice. Starting on day 13, animals received four cycles of intraperitoneal vehicle, paclitaxel, Tislelizumab, or the combination. Tumor growth was monitored every 3 days until day 28, and survival was followed in a parallel cohort. Control tumors grew progressively, whereas paclitaxel alone slowed expansion. The addition of Tislelizumab further reduced tumor weight and volume (Figures 1B–1D). Spleens from paclitaxel-treated mice were enlarged, and the effect was reversed by combination therapy (Figures 1E and 1F). Kaplan-Meier analysis showed that PD-1 blockade plus paclitaxel markedly extended survival compared with either single agent (Figure 1G). Thus, Tislelizumab cooperates with paclitaxel to inhibit 4T1 tumor progression and prolong survival in immunocompetent mice.

**Figure 1:**
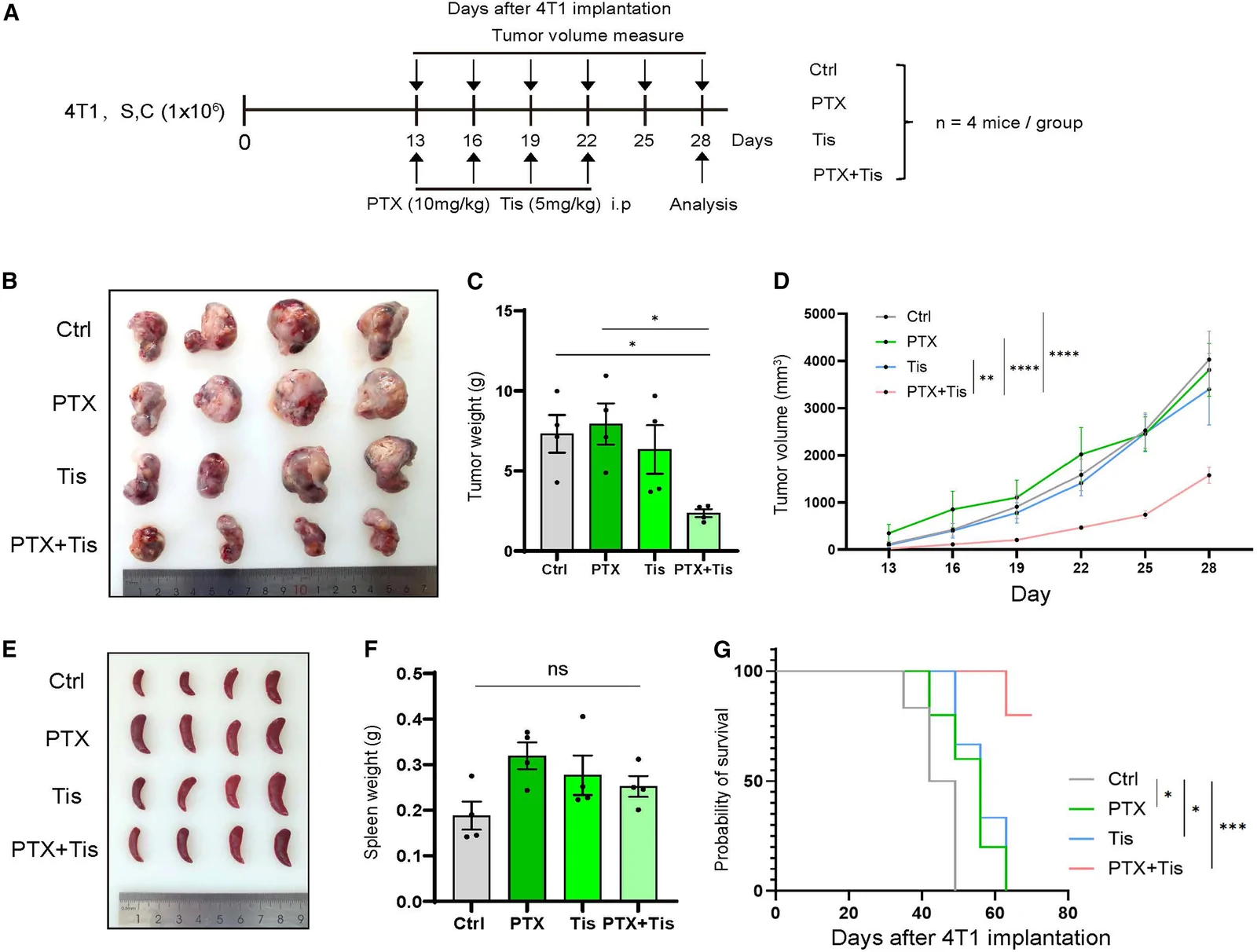
Synergistic antitumor efficacy of paclitaxel and tislelizumab combination therapy in murine 4T1 mammary carcinoma model (A) Immunocompetent syngeneic C57BL/6N mice (6 weeks old, *n* = 4/group) received subcutaneous implantation of 4T1 murine breast cancer cells. Commencing on day 13 post-implantation, animals received intraperitoneal administration of paclitaxel (PTX, 10 mg/kg) or tislelizumab (anti-PD-1 antibody, Tis, 10 mg/kg) as monotherapy or combination therapy (PTX/tislelizumab) on days 13, 16, 19, and 22. Tumor volumes were monitored biometrically every three days starting from day 13 until endpoint at day 28. (B–D) Representative tumor photographs (B), terminal tumor weights (C), and longitudinal tumor growth kinetics (D) demonstrate significant tumor suppression in the combination therapy group. (E and F) Splenic architecture preservation (E) and quantitative splenomegaly analysis (F) indicate enhanced immune homeostasis with combination treatment. (G) Kaplan-Meier curves reveal significantly prolonged survival in the PTX/Tislelizumab-treated cohort (*n* = 5 per/group). Data represent mean ± SEM. ∗*p* < 0.05; ∗∗*p* < 0.01; ∗∗∗*p* < 0.001; and ∗∗∗∗*p* < 0.0001. Two-way ANOVA with Tukey’s multiple comparisons test was applied for tumor growth curves, and one-way ANOVA for terminal tumor weights and spleen weights.

### Paclitaxel and Tislelizumab combination treatment enhances T cell infiltration and apoptosis in tumor tissues

Immunofluorescence profiling of day-28 4T1 tumor revealed that paclitaxel alone modestly increased intratumoral PD-1^+^ cells relative to vehicle controls; the addition of Tislelizumab (PTX + Tis) further elevated PD-1^+^ infiltration (Figures 2A and 2B). CD4^+^ T cell density followed a similar trend, with combination therapy producing the highest signal intensity (Figures 2C and 2D). CD8^+^T cell and granzyme-B (GZMB) co-staining showed sparse positivity in control and single-agent arms, whereas PTX + Tis yielded a pronounced accumulation of GZMB^+^ CD8^+^ effectors at the tumor (Figures 2E and 2F). Western blot (WB) analysis of tumor lysates demonstrated that the anti-apoptotic protein BCL2 declined, while pro-apoptotic BAX and cleaved caspase-3 were markedly induced by PTX + Tis (Figures 2G, 2H, and S4). Concurrently, CXCR5 was up-regulated in the combination group (Figure 2I). Collectively, these data indicate that PD-1 blockade combined with paclitaxel remodels the 4T1 microenvironment toward a cytotoxic, pro-apoptotic state.

**Figure 2:**
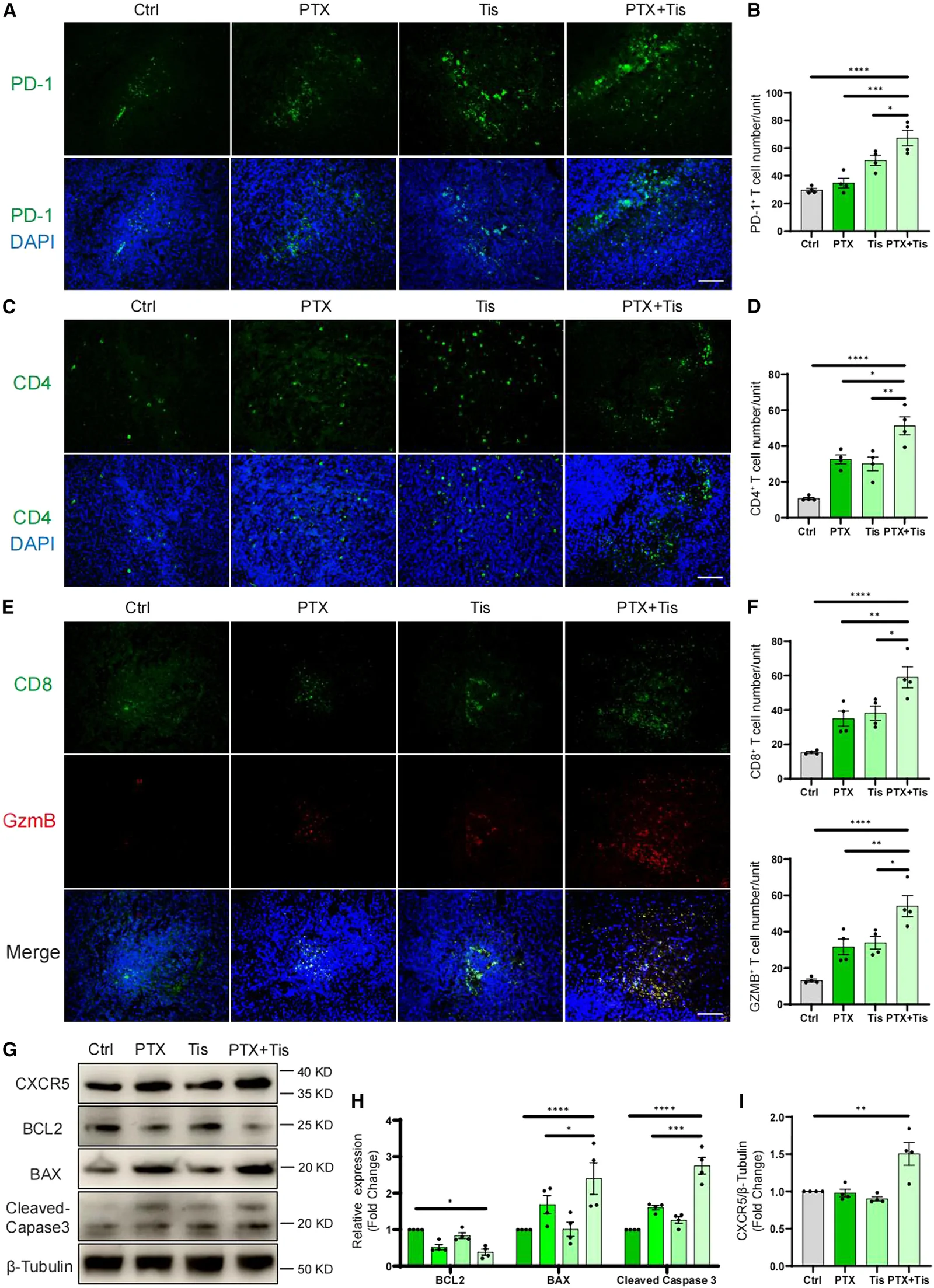
Synergistic effects of paclitaxel and tislelizumab combination therapy on T cell infiltration and apoptosis in 4t1 tumor microenvironment (A–F) Immunofluorescence staining was conducted to evaluate tumor-infiltrating lymphocyte profiles, including (A) PD-1^+^T cells, (C) CD4^+^T helper cells, and (E) CD8^+^ cytotoxic T cells co-expressing granzyme B (GZMB^+^) in 4T1 tumor tissues across experimental groups (*n* = 4 mice per group, 3∼4 tumor image sections per mouse). Representative images show four tissue sections per tumor. Quantitative analysis of (B) PD-1^+^T cell density, (D) CD4^+^T cell infiltration, and (F) CD8^+^/GZMB^+^ T cell co-localization in different treatment cohorts. (G–I) Western blot analysis of apoptotic markers in tumor lysates revealed significant upregulation of pro-apoptotic proteins CXCR5, BAX, and cleaved caspase-3, with corresponding downregulation of anti-apoptotic BCL2 in the PTX/tislelizumab-treated cohort (*n* = 4 independent experiments). Data are presented as mean ± SEM; statistical significance determined by one-way ANOVA for single-factor comparisons and two-way ANOVA for interaction effects, followed by Tukey’s multiple comparisons test: ∗*p* < 0.05; ∗∗*p* < 0.01; ∗∗∗*p* < 0.001; and ∗∗∗∗*p* < 0.0001; Scale bars, 100 μm (applies to all images in A, C, E). GZMB: Granzyme B.

### Overexpression of CXCR5 enhances tumor cell growth inhibition under paclitaxel and Tislelizumab co-treatment

To define the functional contribution of CXCR5 to combination therapy, 4T1 triple-negative breast cancer cells were stably transduced to over-express Flag-tagged CXCR5 or to silence endogenous Cxcr5; empty-vector cells served as controls. WB confirmed robust modulation of CXCR5 protein in both directions (Figures 3A, 3B, and S4). The control, Flag- CXCR5, and ShCxcr5 4T1 cells were implanted subcutaneously into female C57BL/6N mice. Starting on day 13, animals received four cycles of intraperitoneal vehicle, paclitaxel, Tislelizumab, or the combination. Tumor growth was monitored every 3 days until day 28; survival was followed in a parallel cohort (Figure 3C). Consistent with these observations, Cxcr5 knock-down blunted the therapeutic benefit of PTX + Tis, whereas CXCR5 over-expression sensitized tumors to the combination, producing the most pronounced growth inhibition among all experimental arms (Figures 3D–3F). Macroscopic inspection of spleens harvested at day 30 revealed evident splenomegaly in mice bearing Cxcr5 knock-down tumors, whereas CXCR5-over-expressing hosts presented smaller, paler spleens (Figure 3G). Correspondingly, quantitative analysis demonstrated a statistically significant decrease in spleen weight in the Flag-CXCR5 group compared with shCxcr5 cohorts, whereas CXCR5 overexpression yielded the lowest mean splenic mass (Figure 3H). Longitudinal survival monitoring further supported these findings: mice harboring CXCR5-depleted cells succumbed earlier, whereas mice bearing CXCR5-over-expressing tumors experienced markedly extended survival under combination therapy (Figure 3I). Collectively, the data position CXCR5 as a critical, cell-intrinsic determinant that governs both tumor growth restraint and overall therapeutic responsiveness to PD-1 inhibition plus chemotherapy in this aggressive TNBC model.

**Figure 3:**
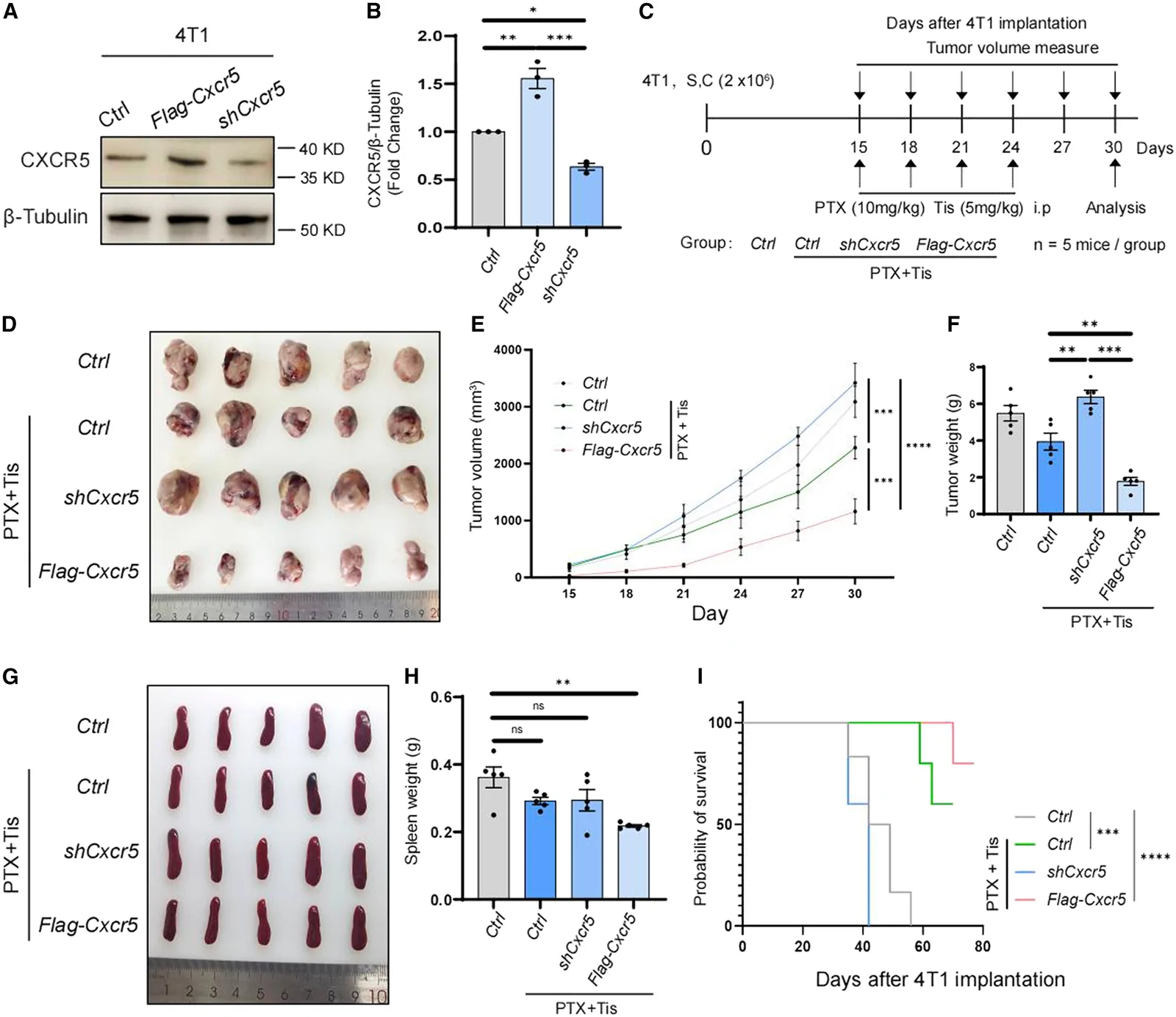
Over expression of CXCR5 potentiates tumor growth inhibition under paclitaxel (PTX) and Tislelizumab (Tis) co-treatment (A and B) Western blot analysis of CXCR5 expression in 4T1 cells transfected with Flag-CXCR5 or shCxcr5 plasmids for 48 h (*n* = 3 independent experiments). (C) Experimental design: 4T1 cells with stable CXCR5 overexpression/knockdown or empty vector controls were subcutaneously injected into 6-week-old C57BL/6N mice (*n* = 5/group). From day 15 post-implantation, mice received intraperitoneal injections of PTX (10 mg/kg), Tis (10 mg/kg), or combination therapy every 3 days (days 15, 18, 21, 24). Tumor volumes were monitored every 3 days until day 30. (D–F) Representative tumor photographs (D), growth kinetics (E), and excised tumor weights (F). (G and H) Spleen morphology (G) and quantified spleen weights (H). (I) Kaplan-Meier survival analysis of tumor-bearing mice (*n* = 5/group; log rank test). Data are presented as mean ± SEM. ∗*p* < 0.05; ∗∗*p* < 0.01; ∗∗∗*p* < 0.001; and ∗∗∗∗*p* < 0.0001 (two-way ANOVA with Tukey’s multiple comparisons test for tumor growth; one-way ANOVA for endpoint measurements).

### Overexpression of CXCR5 enhances T cell infiltration and apoptosis in tumors under paclitaxel and Tislelizumab co-treatment

To understand how tumor-intrinsic CXCR5 shapes the immune microenvironment under PD-1 blockade plus paclitaxel, we performed immunofluorescence on day-30 4T1 tumors. PD-1^+^T cell density was highest in Flag-CXCR5 tumors and lowest in shCxcr5 lesions (Figures 4A and 4B). CD4^+^ infiltration followed an identical pattern (Figures 4C and 4D). Concurrently, CD8^+^ and granzyme-B (GZMB) double-positive cytotoxic T cells were markedly enriched in the Flag-CXCR5 group compared with the empty-vector and shCxcr5 groups (Figures 4E and 4F). WB analysis of whole-tumor lysates revealed that CXCR5 over-expression preserved the anti-apoptotic protein BCL2, whereas CXCR5 knockdown elevated the pro-apoptotic indices BAX and cleaved caspase-3 (Figures 4G, 4H, and S4). Collectively, these data demonstrate that tumor-derived CXCR5 not only recruits PD-1^+^ and CD4^+^ cells but also improves CD8+ effector function and apoptotic signaling, thereby promoting the efficacy of PD-1 inhibition combined with paclitaxel.

**Figure 4:**
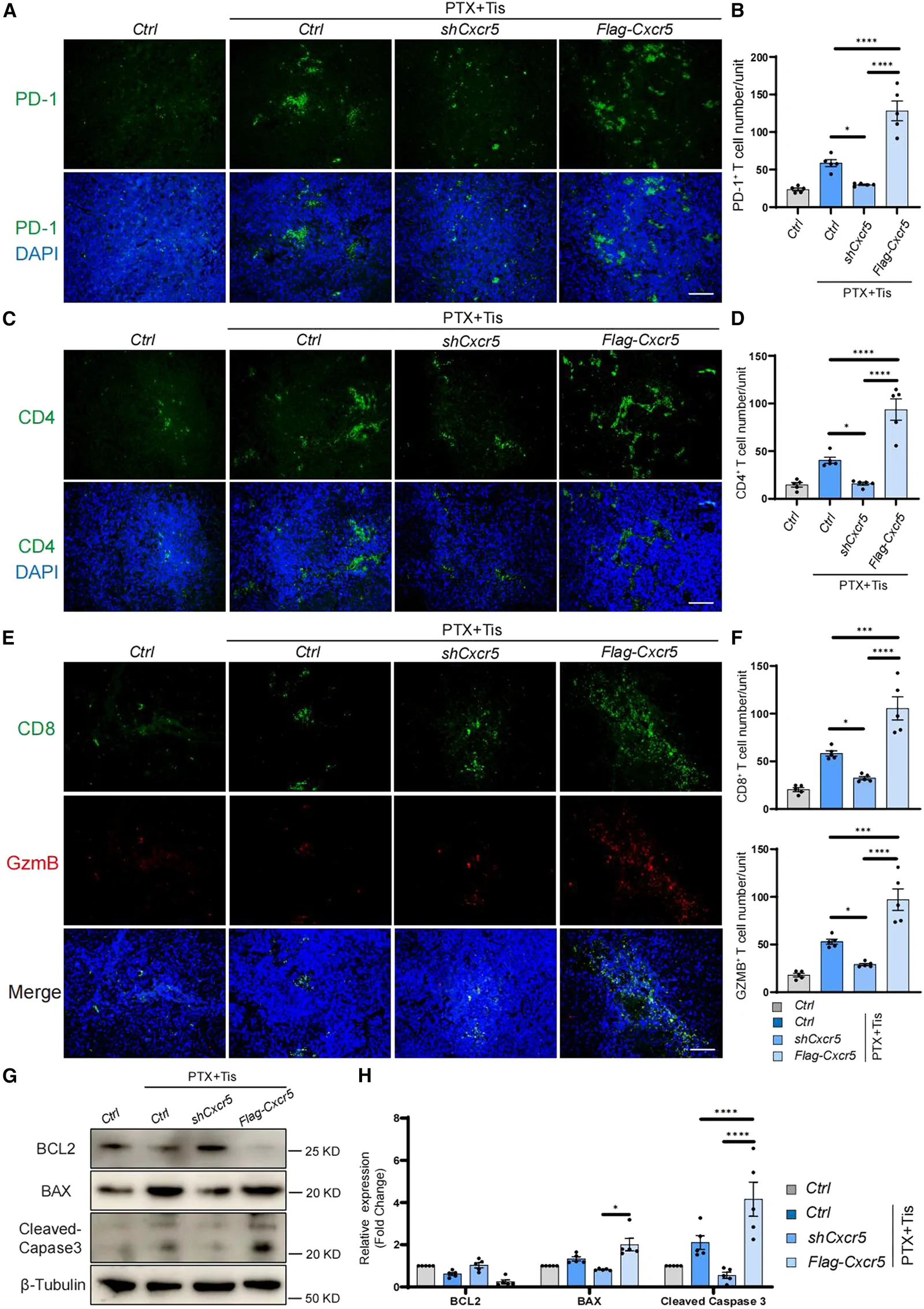
CXCR5 over expression enhances T cell infiltration and tumor cell apoptosis during PTX/Tis combination therapy (A–F) Immunofluorescence analysis of T cell infiltration in 4T1 tumors: (A) PD-1^+^ T cells, (C) CD4^+^ T cells, (E) CD8^+^/GZMB^+^ T cells (*n* = 5 mice per group, 3∼4 tumor image sections per mouse). Quantitative assessment of (B) PD-1^+^, (D) CD4^+^, (F) CD8^+^/GZMB^+^ T cell infiltration. (G and H) Western blot quantification of apoptosis-related proteins (BCL2, BAX, Cleaved Caspase-3) in tumor lysates (*n* = 5). Scale bars, 100 μm (applies to all images in A, C, E). Data are shown as mean ± SEM. ∗*p* < 0.05; ∗∗∗*p* < 0.001; and ∗∗∗∗*p* < 0.0001 (one-way ANOVA with Tukey’s test).

### CXCR5 overexpression modulates the therapeutic efficacy of neoadjuvant immunotherapy combined with chemotherapy in 4T1 tumor cells

Given the pivotal function of CXCR5 in antigen presentation and its positive correlation with CD8^+^ T cell infiltration, we hypothesized that CXCR5 may exert an antitumor effect by potentiating T cell-mediated immune killing. To test this, MDA-MB-231 was engineered to silence endogenous CXCR5 (siCxcr5) or to over-express CXCR5 (Flag-CXCR5), and the expression of CXCR5 was analyzed (Figures 5A–5C and S4). T cells were derived from human peripheral blood mononuclear cells (PBMCs) and activated. CXCR5-silenced or -overexpressing MDA-MB-231 cells were subjected to activated T cells alone, PTX + Tis alone, or the combined treatment. Crystal violet staining revealed that CXCR5-overexpressing cells exhibited a significantly lower survival rate compared to control cells in the presence of T cells or PTX+TIS, indicating enhanced sensitivity (Figures 5D and 5E). To investigate whether CXCR5 enhances T-cell-mediated antitumor effects by promoting chemotaxis, we performed Transwell migration assays using supernatants from CXCR5-overexpressing MDA-MB-231 cells as chemoattractants. Supernatants from Flag-CXCR5 cells significantly increased CD8^+^ T cell migration compared with control supernatants (Figures 5F and 5G), indicating that CXCR5 promotes T cell recruitment. Western blot analysis of the same experimental conditions revealed that CXCR5 overexpression decreased the anti-apoptotic protein BCL2 and elevated pro-apoptotic BAX, thereby priming tumor cells for apoptosis. Conversely, CXCR5-silenced cells maintained high BCL2 and low BAX even after PTX + Tis exposure (Figures 5H, 5I, and S4). Collectively, these *in vitro* data corroborate our *in vivo* findings and establish CXCR5 as a cell-autonomous switch that increase T cell cytotoxicity and promotes the pro-apoptotic program by PD-1 blockade plus chemotherapy.

**Figure 5:**
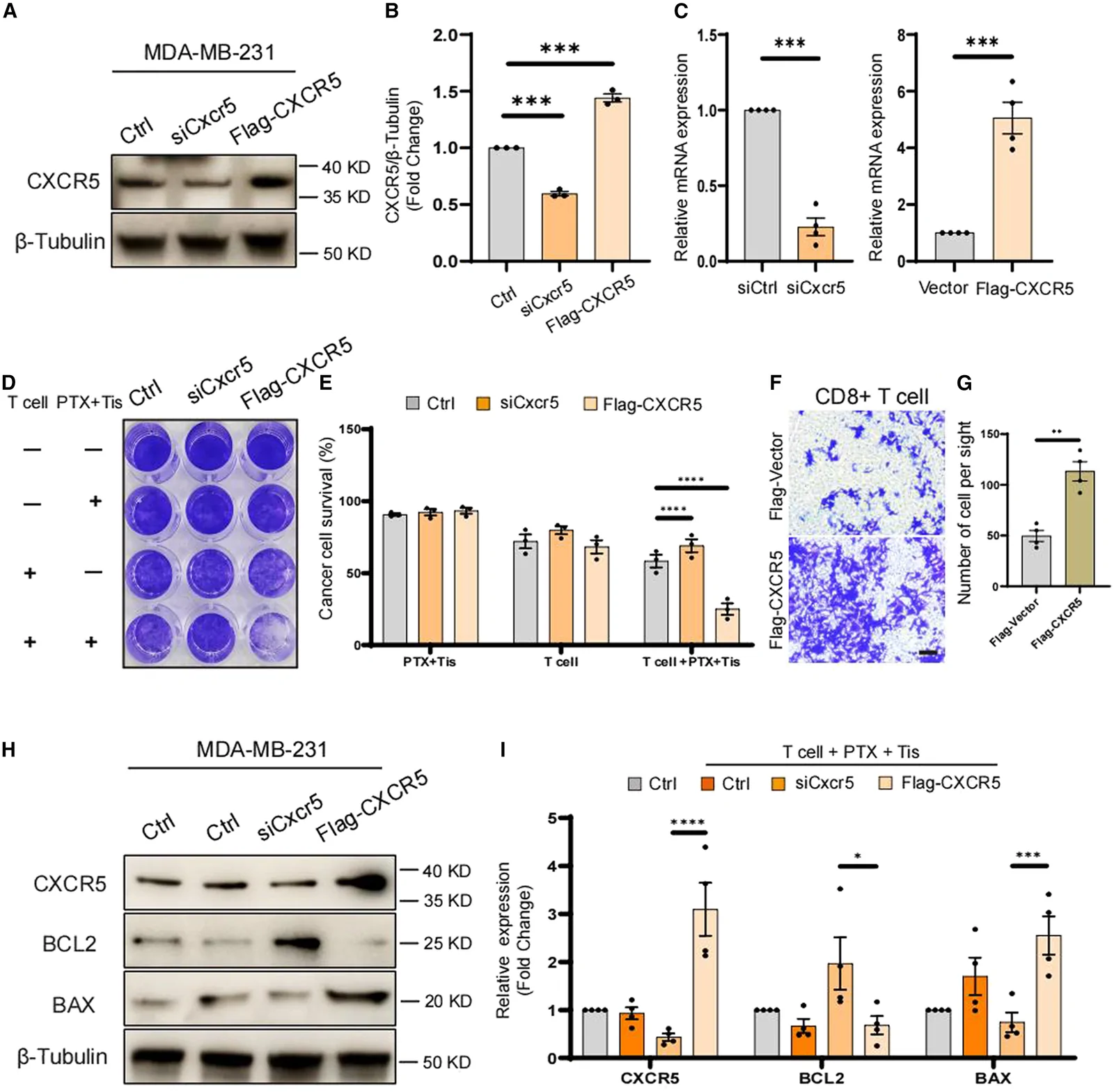
CXCR5 over expression modulates the therapeutic efficacy of neoadjuvant immunotherapy combined with chemotherapy in 4T1 tumor cells (A–C) MDA-MB-231 cells were transfected with CXCR5 siRNA or Flag-CXCR5 plasmid for 48 h. Cellular extracts and total RNA were prepared and analyzed by western blotting (*n* = 3 independent experiments) and qPCR (*n* = 4 independent experiments). (D) T cell-mediated tumor cell killing assay: MDA-MB-231 cells transfected with CXCR5 siRNA or Flag-CXCR5 plasmid were divided into four groups: Ctrl (no treatment), PTX (10 nM), or Tis (10 μg/mL) co-treatment alone, co-cultured with activated T cells for 48 h in the presence or absence of PTX and Tis, followed by crystal violet staining. The effector-to-target ratio (MDA-MB-231: T cell) was 1:5. (E) Quantitative analysis of T cell-mediated cytotoxicity. *n* = 3. (F) Culture supernatants from normal and CXCR5 Overexpression group of tumor cells were added to the lower compartment of the Transwell chamber. CD8^+^ T cells were seeded into the upper chamber. Representative crystal violet images were collected. (G) Quantitative analysis of T cell migration. *n* = 4. (H and I) MDA-MB-231 cells were transfected with CXCR5 siRNA or Flag-CXCR5 plasmid for 48 h. The protein levels of CXCR5, BCL2, and BAX were analyzed by western blot analyzed by western blotting (*n* = 4 independent experiments). Data are shown as mean ± SEM, ∗*p* < 0.05; ∗∗∗*p* < 0.001; and ∗∗∗∗*p* < 0.0001, one-way ANOVA and two-way ANOVA followed by Tukey’s multiple comparisons test. Scale bars, 20 μm (F).

To further assess the functional influence of CXCR5 on chemotherapy sensitivity and immunogenic cell death, we measured cell viability, HMGB1 release, and ATP levels in MDA-MB-231 cells after PTX treatment. As depicted in Figure S2A, no significant difference was detected between CXCR5-overexpressing cells and control cells, suggesting that CXCR5 does not change the intrinsic sensitivity to PTX. PTX-induced HMGB1 release was remarkably enhanced in CXCR5 overexpressing cells and decreased in siCxcr5 cells when compared to the control group (Figure S2B). Similarly, ATP levels, a crucial damage-associated molecular pattern (DAMP) indicating immunogenic cell death, were significantly elevated in CXCR5-overexpressing cells following PTX treatment, while CXCR5 silencing reduced ATP release (Figure S2C). These findings illustrate that CXCR5 potentiates PTX-induced immunogenic cell death (ICD), thus establishing a mechanistic connection between chemotherapy sensitization and immune microenvironment remodeling.

### High expression of CXCL13 and CXCR5 is correlated with a favorable prognosis in patients with TNBC

We compared the pre-treatment tumor RNA-seq profiles between patients who achieved a partial response (PR, *n* = 6) and non-responders (non-PR, *n* = 6) within the PD-1inh + PTX cohort. A total of 15 immune-related genes were differentially expressed (log_2_ fold change > 1.5, FDR < 0.05). PR tumors showed higher expression of IFN-γ signaling molecules (IFNG, CXCL13), cytolytic effectors (GZMK), antigen presentation machinery (B2M, PSMB9, BATF), and PD-L1 (CD274) (Figure S1C). We explored the prognostic value of CXCL13 and CXCR5 in patients with TNBC using the KM plotter. Higher expression levels of both CXCL13 and CXCR5 were significantly associated with prolonged overall survival (OS) in patients with TCGA-TNBC (Figures S1D and S1E), supporting their potential as favorable prognostic biomarkers.

### The CXCL13-CXCR5 axis enhances CD8^+^ T cell migration and modulates apoptosis-related proteins via p38 signaling pathways

To investigate whether CXCR5 enhances T cell-mediated antitumor effects by promoting chemotaxis, we conducted Transwell migration assays. PTX+Tis treatment significantly promoted CD8^+^T cell migration compared to the control group. However, this effect was markedly abolished by the CXCL13 inhibitor VX5 (Figures 6A, 6B, and S4), indicating that PTX+Tis-induced T cell recruitment is dependent on CXCL13/CXCR5 signaling.

**Figure 6:**
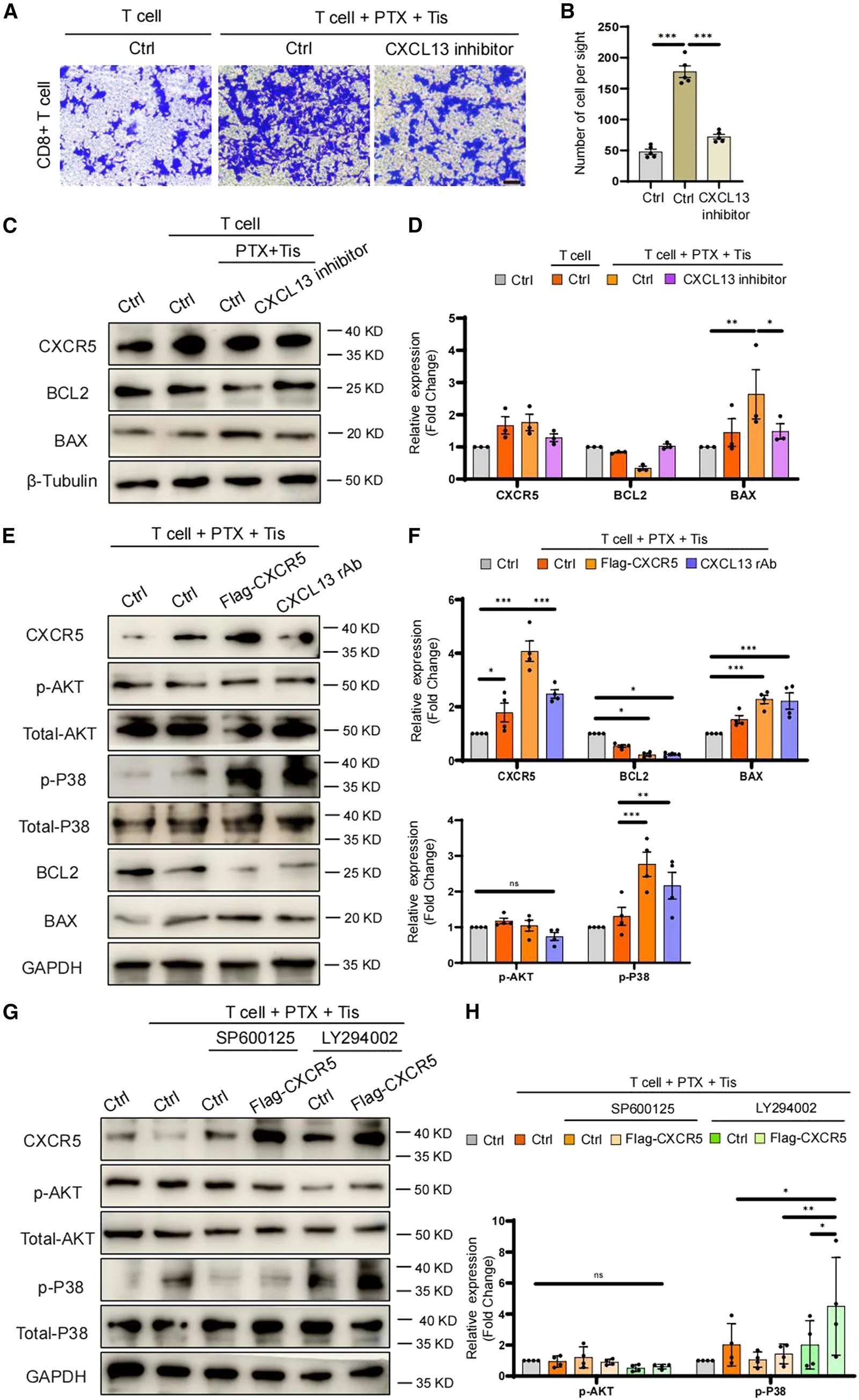
Tumor intrinsic CXCR5 enhances the migration of CD8^+^ T cells and modulates apoptosis-related proteins through CXCL13-dependent signaling (A and B) Representative images and quantitative analysis of the Transwell migration assay. The migration of CD8^+^T cells induced by PTX+Tis is abolished by the CXCL13 inhibitor VX5 (*n* = 4). (C and D) Western blot analysis and quantification of CXCR5, BCL2, and BAX in MDA-MB-231 cells under the specified conditions (*n* = 3). (E and F) Western blot analysis and quantification of CXCR5, BCL2, BAX, p-AKT, and p-p38 in CXCR5-overexpressing/CXCL13 recombinant protein-treated cells, with or without PTX + Tis (*n* = 4). (G and H) Western blot analysis and quantification of CXCR5, BCL2, BAX, *p*-AKT, and p-p38 in cells treated with an AKT inhibitor (LY294002) or a JNK/p38 pathway inhibitor (SP600125) (*n* = 3). The data are presented as the mean ± SEM. ∗*p* < 0.05; ∗∗*p* < 0.01; and ∗∗∗*p* < 0.001; ns, not significant. One-way or two-way ANOVA with Tukey’s test was used for statistical analysis. Scale bars, 20 μm (A).

Next, we examined the role of the CXCL13/CXCR5 axis in modulating the expression of apoptosis-related proteins. Western blot analysis revealed that PTX + Tis treatment upregulated CXCR5 expression and shifted the BCL2/BAX balance toward a pro-apoptotic state, while the CXCL13 inhibitor reversed these effects (Figures 6C, 6D, and S4). Moreover, treatment with CXCL13 recombinant protein (CXCL13 rAb) in combination with PTX+Tis further enhanced CXCR5 expression, reduced BCL2 levels, and increased BAX levels, accompanied by the activation of p38 signaling pathways (Figures 6E, 6F, and S4). To dissect the downstream signaling pathways, we treated CXCR5-overexpressing cells with the AKT inhibitor LY294002 or the JNK/p38 pathway inhibitor SP600125. Inhibition of the JNK/p38 pathway attenuated the CXCR5-mediated downregulation of BCL2 and upregulation of BAX, suggesting that JNK/p38 signaling is required for CXCR5-modulated apoptotic priming (Figures 6G, 6H, and S4).

## Discussion

This study establishes that integrating PD-1 checkpoint blockade with taxane-based chemotherapy elicits superior clinical outcomes in triple-negative breast cancer (TNBC), and uncovers a cell-autonomous requirement for CXCR5 in orchestrating this therapeutic synergy. CXCR5-dependent chemotactic signaling potentiates tumor-infiltrating lymphocyte (TIL) accumulation, amplifies cytotoxic effector programs, and promotes tumor cell apoptosis through caspase cascade induction. Conversely, CXCR5 ablation compromises therapeutic efficacy by impairing T cell trafficking. Collectively, these findings position CXCR5 as a pivotal immunometabolic checkpoint that coordinates antitumor immunity and checkpoint blockade sensitivity.

Clinically, we observed that patients receiving paclitaxel plus a PD-1 inhibitor had significantly prolonged OS and PFS compared to those receiving paclitaxel alone. This is consistent with large-scale trials such as KEYNOTE-355, which demonstrated improved outcomes with the addition of pembrolizumab to chemotherapy in PD-L1–positive TNBC.^2^ Importantly, our transcriptomic analysis revealed that responders to the combination therapy exhibited elevated expression of IFN-γ signaling genes, cytolytic molecules (GZMK), and CXCL13, a chemokine that recruits CXCR5^+^ immune cells. These data support the notion that effective antitumor immunity requires a pre-existing or therapy-induced immune-active tumor microenvironment (TME), as previously reported in melanoma and lung cancer.^2^^,^^3^^,^^20^

Mechanistically, our findings highlight CXCR5 as a tumor-intrinsic determinant of immunotherapy response. In both murine and human TNBC models, CXCR5 overexpression enhanced the recruitment of CD8^+^ and CD4^+^ T cells, increased GZMB production, and promoted tumor cell apoptosis under combination therapy. Importantly, we provide new evidence that tumor-intrinsic CXCR5 enhances chemosensitivity and immunogenic cell death. PTX-induced HMGB1 release and ATP levels were markedly increased in CXCR5-overexpressing cells and diminished in siCxcr5 cells (Figure S2). These DAMPs are key triggers of antitumor immunity, suggesting that CXCR5 potentiates both direct chemotherapeutic and immune-mediated effects. Furthermore, using CXCL13 neutralization and recombinant protein, we showed that the CXCL13-CXCR5 axis is required for PTX+Tis-induced CD8^+^ T cell migration and apoptotic priming. Mechanistically, CXCR5 signaling activated p38 MAPK, and inhibition of p38 abrogated CXCR5-mediated BCL2 downregulation and BAX upregulation, identifying p38 as a critical downstream effector of CXCR5-driven apoptosis. These findings are consistent with findings that CXCL13-producing CD8^+^ T cells can directly induce tumor cell death in hepatocellular carcinoma,^18^ and previously reported the dual role of CXCR5 signaling in promoting both immune activation and tumor cell apoptosis depending on context.^21^ Recent single-cell studies show that CXCR5^+^ CD8^+^ T cells exhibit superior cytotoxicity and proliferation potential^22^ and that CXCL13–CXCR5 interactions are essential for TLS formation and antigen-specific T cell activation.^15^^,^^16^

TLS and immune cell spatial organization in determining immunotherapy response. IF staining revealed that CXCR5 overexpression was associated with increased infiltration of PD-1^+^, CD4^+^, and CD8^+^ T cells, as well as elevated granzyme B expression and CXCL13 levels (Figures S3A and S3B). These findings are consistent with the latest research advances that TLS, often marked by CXCR5 expression, are predictive biomarkers of immunotherapy response in multiple solid tumors, including melanoma, lung cancer, and breast cancer.^15^^,^^16^^,^^17^ Furthermore, our results align with evidence that CXCR5-related gene signatures are not only prognostic but also predictive of response to immune checkpoint inhibitors across multiple cancer types. For instance, in muscle-invasive bladder cancer, high intratumoral CXCL13 expression was associated with improved survival not only in patients receiving immunotherapy, but not in those treated with chemotherapy alone.^23^ Similarly, in metastatic melanoma, the presence of CXCR5^+^ CD8^+^ T cells within TLS was strongly correlated with response to anti-PD-1 therapy and prolonged survival.^24^ These observations reinforce the idea that CXCR5 is a conserved and dynamic biomarker of immune activation that can be leveraged for patient stratification and treatment optimization.

It is worth noting that the exploration of tumor-intrinsic CXCR5 in this study opens up a new perspective compared to previous research that primarily concentrated on CXCR5 expressed on immune cells such as T follicular helper (Tfh) cells and CD8+T cells.^25^^,^^26^ We hypothesize that, on both immune cells and tumor cells, CXCR5 may respond to its ligand CXCL13. This shared ligand -receptor interaction might lead to some overlapping functions. In both cases, CXCR5/CXCL13 signaling can trigger chemotaxis. In the tumor microenvironment, tumor-secreted CXCL13 might attract CXCR5-positive immune cells, while tumor cells themselves can also respond to the secreted CXCL13, which could potentially create a complex interplay between the tumor and the immune system. Regarding the possibility of an autocrine feedback loop, it is feasible that tumor cells with CXCR5 can produce CXCL13. This would allow the tumor cells to stimulate themselves through the CXCR5/CXCL13 interaction. Such an autocrine loop could continuously activate intracellular signaling pathways within the tumor cells, promoting their survival and proliferation. As for other possible mechanisms, reverse signaling could be a potential way for CXCR5-expressing tumor cells to function.^27^ Reverse signaling occurs when the ligand not only activates the receptor but also transduces signals back into the ligand-expressing cell. In the context of CXCR5/CXCL13, if CXCL13-expressing cells interact with CXCR5 on tumor cells, the tumor cells might send signals back to the CXCL13-producing cells, influencing their behavior. However, further research is needed to fully understand the distinct and overlapping functions of CXCR5 on tumor cells compared to immune cells, as well as to clarify the exact mechanisms, such as the autocrine feedback loop and reverse signaling.

Directly targeting tumor-intrinsic CXCR5 may provide a high level of specificity. Tumor cells often express CXCR5 in a distinct pattern compared to normal cells. By focusing on CXCR5 on tumor cells, we can potentially avoid off-target effects on immune cells that also express CXCR5, which may lead to fewer side effects associated with the disruption of normal immune function that may occur when targeting the microenvironmental CXCL13/CXCR5 axis. Tumor intrinsic CXCR5 may be involved in unique signaling pathways that are crucial for tumor growth, survival, and metastasis. Directly targeting this receptor on tumor cells may possibly block these tumor-specific signaling cascades. While targeting the microenvironmental CXCL13/CXCR5 axis may potentially modulate the entire tumor microenvironment, including immune cell recruitment and function. However, there are some challenges of directly targeting tumor Cell CXCR5. Firstly, tumor cells are highly heterogeneous, and the expression levels of CXCR5 can vary significantly among different tumor cells within the same tumor and across different patients. This heterogeneity makes it difficult to develop a universal dosing strategy for the target. Secondly, tumors may develop resistance to CXCR5 targeting agents by altering their signaling pathways or downregulating CXCR5 expression, which may limit the long-term effectiveness of the treatment. Thirdly, ensuring the effective delivery of CXCR5 targeting agents to tumor cells can be a challenge. Importantly, a well-designed clinical translation pathway, this approach may offer a promising approach for cancer treatment, but further research is needed to fully understand its potential and limitations.

Studies have shown that the CXCL13/CXCR5 axis not only activates the classical PI3K/AKT pathway but also engages the JNK and p38 MAPK pathways. In neuroinflammation models, CXCL13/CXCR5 signaling promotes pro-inflammatory cytokine production (TNF-α, IL-6, IL-1β) and immune cell infiltration through JNK and p38 activation,^28^ indicating that CXCR5 possesses the capacity to activate these pathways. Notably, chemokine receptors such as CCR5 and CXCR4 can signal through G-protein-independent mechanisms to activate JNK and p38 MAPK cascades, leading to AP-1 activation and downstream gene regulation.^29^ This provides a mechanistic basis for CXCR5 to preferentially activate JNK/p38 over PI3K/AKT under certain conditions. Direct Phosphorylation and Activation of Bax by JNK and p38 Bax is a key pro-apoptotic protein whose activation and mitochondrial translocation are critical steps in the mitochondrial apoptotic pathway. Kim et al. demonstrated that JNK and p38 directly phosphorylate Bax, as shown by isoelectric point shift analysis and [^32^P] metabolic labeling.^30^ This phosphorylation is required for Bax activation and mitochondrial translocation. Inhibition of JNK/p38 or silencing of MAPKK4 (an upstream kinase) blocked Bax phosphorylation, mitochondrial translocation, and apoptosis. Confocal microscopy further identified Thr167 as the critical phosphorylation site, as the T167D mutant failed to translocate to mitochondria.^30^ Synergistic Amplification via the JNK/Bim/Bax Cascade Beyond direct Bax phosphorylation, JNK can indirectly promote Bax activation through the pro-apoptotic protein Bim. In UV-induced apoptosis models, JNK activation led to Bim mitochondrial translocation and subsequent Bax activation, events blocked by the JNK inhibitor SP600125.^31^ These findings suggest that when CXCR5 preferentially activates JNK, it can promote Bax activation and mitochondrial translocation through two synergistic mechanisms-the “JNK-Bim-Bax” cascade and direct “JNK/p38-Bax” phosphorylation-thereby efficiently initiating the mitochondrial apoptotic pathway. To explore the molecular mechanism by which CXCR5 regulates CD8+T cell infiltration and tumor cell apoptosis, we performed pathway-specific inhibition experiments. Treatment with CXCL13 recombinant protein (CXCL13 rAb) in combination with PTX+Tis further enhanced CXCR5 expression, reduced BCL2, and increased BAX levels, accompanied by activation of p38 signaling pathways (Figures 6E and 6F). To dissect the downstream signaling, we treated CXCR5-overexpressing cells with the AKT inhibitor LY294002 or the JNK/p38 pathway inhibitor SP600125. Inhibition of the JNK/p38 pathway attenuated the CXCR5-mediated downregulation of BCL2 and upregulation of BAX, suggesting that JNK/p38 signaling are required for CXCR5-modulated apoptotic priming (Figures 6G and 6H). Furthermore, using CXCL13 neutralizing antibodies in migration assays or verifying the p38/Bax signaling axis in *in vivo* tumor models would further confirm the results.

In summary, we establish CXCR5 as a tumor-intrinsic molecular switch that confers synergistic sensitivity of metastatic TNBC to paclitaxel-plus-PD-1 blockade by coordinating T cell recruitment and tumor-cell-intrinsic apoptosis. Targeting CXCR5 therefore may represent a precision-immunology lever. Future studies exploring “transient CXCR5 agonism” strategies-such as engineered CXCL13-Fc fusion proteins or tumor-selective CXCR5-encoding mRNA-lipoplexes-as neoadjuvant interventions to convert immunologically “cold” TNBC into T-cell-inflamed tumors are in demand.^32^^,^^33^ Longitudinal monitoring of circulating CXCR5 levels or CXCR5^+^ T cell dynamics in peripheral blood may additionally serve as a minimally invasive biomarker of early response or emerging resistance.

Contextualizing these findings above, the CXCR5 signaling in metastatic TNBC warrants rigorous investigation as both a predictive biomarker and a therapeutic target within taxane-PD-1 inhibitor regimens. Context-specific modulation of this chemokine-receptor dyad emerges as a promising strategy to overcome adaptive resistance through enhancing tertiary lymphoid structure formation and potentiating CD8^+^ T cell metabolic fitness during combination immunotherapy.

### Limitations of the study

There are several limitations of the present study. First, the study lacks detection of CXCL13 levels and blocking experiments to validate this central axis, reflecting an insufficient elucidation of specific downstream signaling pathways by which CXCR5 regulates apoptosis and immune recruitment. Secondly, the study has a small, retrospective clinical sample size. Thirdly, animal models using immunocompetent mice may not fully recapitulate the complexity of the human tumor microenvironment. Fourth, the chemokine network exhibits functional redundancy, with alternative axes such as CXCR3-CXCL9/10 potentially compensating for CXCR5 loss. Fifth, other anti-apoptotic family members, including MCL1 and BCL-XL, may sustain survival signals when BCL2 is downregulated. Sixth, the specificity of CXCR5 as a single biomarker may be limited, suggesting that a multi-gene signature incorporating CXCR5 and its potential compensatory partners could improve predictive accuracy. Seventh, there is a lack of examination of the potential existence of compensatory mechanisms in CXCR5-low but responsive patients due to the limited number of patients with low CXCR5 expression who responded to the combination therapy. Seventh, lack of data on spatial colocalization of CXCR5+ tumor cells and CD8^+^ T cells due to insufficient samples. Lastly, lack of rescue experiments using a BCL2 overexpression plasmid to confirm whether the enhanced therapeutic sensitivity by CXCR5 indeed depends on the BCL2/BAX signaling.

## Resource availability

### Lead contact

Further information and requests for resources and reagents should be directed to and will be fulfilled by the lead contact Fenge Li, E-mail: rosetea85@163.com, +8613821072072, mail to Jieyuan Road No.190, Hongqiao District, Tianjin, China 300121.

### Materials availability

Materials used in this study are available from the corresponding author upon reasonable request.

### Data and code availability

• Raw data can be provided by the corresponding authors upon reasonable request.
• Code availability not applicable.
• Any additional information required to reanalyze the data reported in this paper is available from the lead contact upon request.

## Acknowledgments

This work was supported by the District-level Research Project of Tianjin Bichen District Health Commission (grant nos SHGY-2024008 and SHGY-2021003). This study was also supported by the key research project funded by the First Affiliated Hospital of Nankai University key funding project (funding no 2025YJZD002), the 10.13039/501100006606Natural Science Foundation of Tianjin established by 10.13039/501100004945Nankai University independently (no.:24JCYBJC01590), the Tianjin Medical Key Discipline Construction funding (no TJYXZDXK-3-003A-5). This work was also supported by the general research projects funded by the First Affiliated Hospital of Nankai University (funding nos 2024YJ023, 2019ZGJCJJLM and 2024YJ020).

## Author contributions

Conceptualization, W.F., J.Y., X.G., and F.L.; methodology, F.L. and J.H.; software, W.F. and J.H.; validation, F.L. and J.H.; formal analysis, W.F., J.Y., F.L., J.H., and M.J.; investigation, X.G. and J.Y.; resources, W.F., J.Y., X.G., and F.L.; data curation, W.F., J.Y., F.L., J.H., M.J., and X.L.; writing – original draft preparation, W.F., J.Y., F.L., J.H., and M.J.; writing-review and editing, F.L.; visualization, C.M.; supervision, C.M.; project administration, F.L. and C.M.; funding acquisition, W.F., J.Y., F.L., and X.G. The co-first authors (W.F., J.Y., and X.G.) contributed equally and have the right to list their names first in their CV.

## Declaration of interests

The authors declare no competing interests.

## STAR★Methods

### Key resources table

**Table:** 

REAGENT or RESOURCE	SOURCE	IDENTIFIER
**Antibodies**		

anti-CXCR5 antibody	Proteintech	Cat#28239-1-AP; RRID: AB_3086040
anti-BAX antibody	Proteintech	Cat#50599-2-Ig; RRID: AB_2061561
anti-BCL2 antibody	Proteintech	Cat#26593-1-AP; RRID: AB_2818996
anti-Cleaved Caspase 3	Proteintech	Cat#25128-1-AP: RRID: AB_3073913
anti-Phospho-AKT	Proteintech	Cat#28731-1-AP; RRID: AB_2881201
anti-p38 MAPK	Proteintech	Cat#14064-1-AP; RRID: AB_2878007
anti-Phospho-p38 MAPK	Proteintech	Cat#28796-1-AP; RRID: AB_2918205
GAPDH	Utibody	Cat#UM4002; RRID: AB_3665266
β-Tubulin	Utibody	Cat#UM4003; RRID: AB_3665265
anti-CD8A antibody	Thermo Fisher Scientific	Cat#14–0081–82; RRID: AB_467087
anti-Granzyme B antibody	Proteintech	Cat#13588-1-AP; RRID: AB_2114429
anti-CD4 antibody	Proteintech	Cat#19068-1-AP; RRID: AB_10603357
anti-PD-1antibody	Proteintech	Cat#66220-1-Ig; RRID: AB_2881611
anti-CD3 antibody	Thermo Fisher Scientific	Cat#16–0037; RRID: AB_468855

**Experimental models: Cell lines**		

MDA-MB-231	Procell	Cat#CL-0150
4T1	Procell	Cat#CL-0007

**Experimental models: Organisms/strains**		

C57BL/6N, mouse	Beijing Vital River Laboratory Animal Technology Co., Ltd.	N/A

**Other**		

ImmunoCult Human CD3/CD28/CD2 T cell activator	STEMCELL Technologies	10970
Tislelizumab	BeOne Medicines Ltd.	N/A

### Experimental model and study participant details

#### Ethics statement

This study conformed to the ethical guidelines of the Declaration of Helsinki and was approved by The Tianjin Beichen Hospital.The ethical approval number for the animals used in the study is No. 2024121908.

#### Animal models establishment

Six-week-old male immunocompetent C57BL/6N mice were obtained from Beijing Vital River Laboratory Animal Technology Co., Ltd. and maintained under specific pathogen-free conditions at the Laboratory Animal Center of Tianjin Beichen Hospital. All animal procedures were conducted in accordance with institutional guidelines and were approved by the Ethics Committee of Tianjin Beichen Hospital.

Two independent sets of tumor-bearing mouse models were established for this study. In the first experimental cohort, 4T1 cells were subcutaneously injected into four groups of mice (four mice per group) at a density of 1 × 10^6^ cells per mouse. Tumor growth was monitored beginning on day 13 post-inoculation, with tumor volume measurements recorded every three days. 13 days post-inoculation, the mice received one of the following treatments: phosphate-buffered saline (PBS), TIS monotherapy (10 mg/kg), paclitaxel (PTX) monotherapy (10 mg/kg), or a combination of TIS and PTX. All drugs were administered intraperitoneally on days 13, 16, 19, and 22. On day 28, all mice were euthanized, and tumor tissues and spleens were harvested for subsequent analyses.

In the second experimental cohort, the three previously mentioned 4T1 cells, which were stably transfected with an empty vector control, CXCR5 targeting shRNA, or a CXCR5 overexpressing plasmid, were subcutaneously injected into four groups of mice (with five mice in each group). Specifically, two groups were injected with the cells transfected with the empty vector control, one group received the shCxcr5 knockdown cells, and one group was injected with the CXCR5 overexpressing cells. 15 days post-inoculation, the mice received one of the following treatments: PBS or a combination of TIS and PTX. All drugs were administered intraperitoneally on days 15, 18, 21, and 25. Tumor volume measurements commenced on day 15 and were recorded every three days thereafter. On day 31, all animals were euthanized, and tumor tissues and spleens were collected for further analysis. Tumor volume was calculated using the formula: length×width^2^/2, where length was the longest diameter and width was the shortest diameter.

#### Cell culture and regents

The human triple-negative breast cancer cell line MDA-MB-231 (Procell, CL-0150) and the murine triple-negative breast cancer cell line 4T1 (Procell, CL-0007) were maintained in Leibovitz’s L-15 (PM151010) or RPMI-1640 (PM150110) supplemented with 10% fetal bovine serum (FBS; Gibco; Cat. No. 10099141) and 1% penicillin–streptomycin (Gibco; Cat. No. 15140122). The cell lines used in the study have been authenticated using morphological examination and growth characteristics analysis and short tandem repeats (STR) profiling. The cell lines were also tested for mycoplasma contamination. Regent: Human CXCL13 inhibitor (VX5, HY-P991567, MCE), Human CXCL13 recombinant protein (BCA1-1/CXCL13, HY-P7131, MCE). AKT inhibitor (LY294002, S1105, Selleck), JNK/P38 inhibitor (SP600125, S1460, Selleck).

### Method details

#### Plasmids

Short hairpin RNAs (shRNAs) of CXCR5 were procured from Sangon Biotech (Shanghai, China), while plasmids containing CXCR5 cDNA, used to generate stable CXCR5 overexpressing human and mouse cells, were obtained from Sino Biological (Beijing, China). The shRNA sequences: Human CXCR5-F CACGTTGCACCTTCT CCC AA; Human CXCR5-R GGAATCCCGCCACATGGTAG.

#### siRNA and plasmid transfection

Cells were seeded into plates at 80% confluence. For siRNA transfection, cells were transfected with control siRNA or gene-specific siRNA (100 nM) using Lipofectamine RNAi MAX transfection reagent (Thermo Fisher Scientific, #13778–150) according to the manufacturer’s instructions. For plasmid transfection, Lipofectamine 2000 (Thermo Fisher Scientific, Waltham, #11668027) were used according to the manufacturer’s instructions. Fresh medium was added 6 h after transfection. Gene-specific siRNA sequences: Human CXCR5-siRNA CUGGACAGAUUGGACAACU.

#### RNA extraction and quantitative RT- PCR analysis

For *in vitro* cultured cells, the cells were harvested for RNA extraction using TRIzol reagent. RNA concentration was determined by spectrophotometry using a Nanodrop ND-1000 (Thermo Fisher Scientific, USA), and RNA samples were reverse-transcribed to complementary DNA (cDNA) using TransScript One-Step gDNA Removal and cDNA Synthesis SuperMix (TransGen, #AT311-02). qPCR was performed using PerfectStartTM Green qPCR SuperMix (TransGen, #AQ602-14/24) and was processed with QuantStudio 5 Real-Time PCR system (Applied Biosystems, United States). β-actin was used as internal control. All qPCR results were obtained from at least three biological replicates.

#### Western blot (WB)

Whole-cell lysates were prepared with RIPA buffer ((20 mM NAP, pH7.4, 150 mM NaCl, 1% Triton, 0.5% Sodium-deoxycholate, and 0.1% SDS) containing protease inhibitor. The lysates were sonicated and centrifuged at 12,000 × g for 15 min at 4°C and subjected to protein concentration using BCA Assay Reagent (Thermo Fisher Scientific). The proteins were denatured and resolved by SDS–PAGE and immunoblotted with primary/second antibodies as follows: CXCR5 (28239-1-AP, 1:1000, Proteintech), BAX (50599-2-Ig, 1:1000, Proteintech), BCL2 (26593-1-AP, 1:1000, Proteintech), Cleaved Caspase 3 (25128-1-AP, 1:1000, Proteintech); AKT (10176-2-AP, 1:1000, Proteintech), Phospho-AKT (Ser473) (28731-1-AP, 1:1000, Proteintech), p38 MAPK (14064-1-AP, 1:1000, Proteintech), Phospho-p38 MAPK (Thr180/Tyr182) (28796-1-AP, 1:1000, Proteintech), GAPDH (UM4002, 1:1000, Utibody), β-Tubulin (UM4003, 1:1000, Utibody).

#### Immunofluorescence (IF)

For tissue immunofluorescence, paraffin tumor tissue sections from syngeneic mouse model were baked at 60°C for 2 h, followed by deparaffinization and rehydration in a series of graded alcohols until water was used. Antigen retrieval was performed by submerging the tissue samples into boiling sodium citrate buffer (10 mM, pH 9.0), maintaining for 5 min, three times. Tissue samples were blocked with BSA Blocking Buffer for 1 h at room temperature. Primary antibodies targeting CD8A (#14–0081–82, 1:100, Thermo Fisher Scientific), Granzyme B (13588-1-AP, 1:100, Proteintech), CD4 (19068-1-AP, Proteintech) and PD-1 (66220-1-Ig, 1:100, Proteintech) were added to the tissue slides and incubated overnight at 4°C in a humidified chamber. After extensive washing with PBS, fluorescent-dye conjugated secondary antibodies were added to the tissue slides and incubated for 45 min at room temperature. DAPI was used for nuclei counterstaining.

#### T Cell-mediated tumor cell killing assay

MDA-MB-231 cell lines were cultured in ImmunoCult-XF T cell expansion medium (10981; STEMCELL Technologies). ImmunoCult Human CD3/CD28/CD2 T cell activator (10970; STEMCELL Technologies) and IL-2 (10 ng/mL; PeproTech, Rocky Hill, NJ, USA) were added to the culture medium and maintained for one week following the manufacturer’s protocols. The activated T cells were maintained in DMEM/F12 medium supplied with anti-CD3 antibody (100 ng/mL; 16–0037; Thermo Fisher Scientific) and IL-2 (10 ng/mL) co-cultured with MDA-MB-231 cells for 24 h. After extensive washing with PBS, the living tumor cells were quantified by a spectrometer at OD (570 nm) followed by crystal violet staining.

#### Cell migration assay

MDA-MB-231 cells were seeded into the lower compartment of the Transwell chamber. In accordance with the experimental grouping, PTX, Tis, or the CXCL13 inhibitor VX5 (at a concentration of 10 μg/mL) was added to pretreat the tumor cells for 30 min. The culture supernatants were collected from the normal and CXCR5 overexpression groups of tumor cells and added to the lower compartment of the Transwell chamber as a source of chemoattractant. CD8^+^T cells isolated from the peripheral blood mononuclear cells (PBMCs) of healthy donors were seeded into the upper chamber. After incubation at 37°C in a 5% CO_2_atmosphere for 12–24 h, the upper chamber was gently removed. Subsequently, the cells that had migrated to the lower compartment were stained with a crystal violet solution for 10–20 min. Then, the number of purple stained nuclei or intact cells per microscopic field was counted.

### Quantification and statistical analysis

All statistical analyses were conducted using GraphPad Prism software (version 10.4.1). Data are expressed as mean ± standard error of the mean (SEM). To calculate the statistical significance of differences between two groups, Two-tailed unpaired Student’s *t* test was used. One-way ANOVA and two-way ANOVA followed by Tukey’s multiple comparisons test were used to determine the statistical significances three or more groups. A value of *p* < 0.05 was considered significant. ∗*p* < 0.05, ∗∗*p* < 0.01, ∗∗∗*p* < 0.001, ∗∗∗∗*p* < 0.0001. Mice were randomly assigned for different treatment. No animals were excluded from the study. All experimenters were blinded both during the execution and analysis of experiments.
