# Supplementary material for: CXCR5 regulates CD8+/GZMB+ T cell infiltration and apoptotic signaling in breast cancer
Source: iScience. 2026 Oct 21;29(11):117874. doi: 10.1016/j.isci.2026.117874 (PMC13717742; doi:10.1016/j.isci.2026.117874)
Supplement: Supplementary file 1 [file mmc1.pdf]

iScience, Volume *29*

Supplemental information

CXCR5 regulates $CD8^{+}$/$GZMB^{+}$ T cell infiltration and apoptotic signaling in breast cancer

Weihong Feng, Jin Yan, Xue Gong, Jiandong Hu, Mengli Jin, Chunhua Ma, and Fenge Li

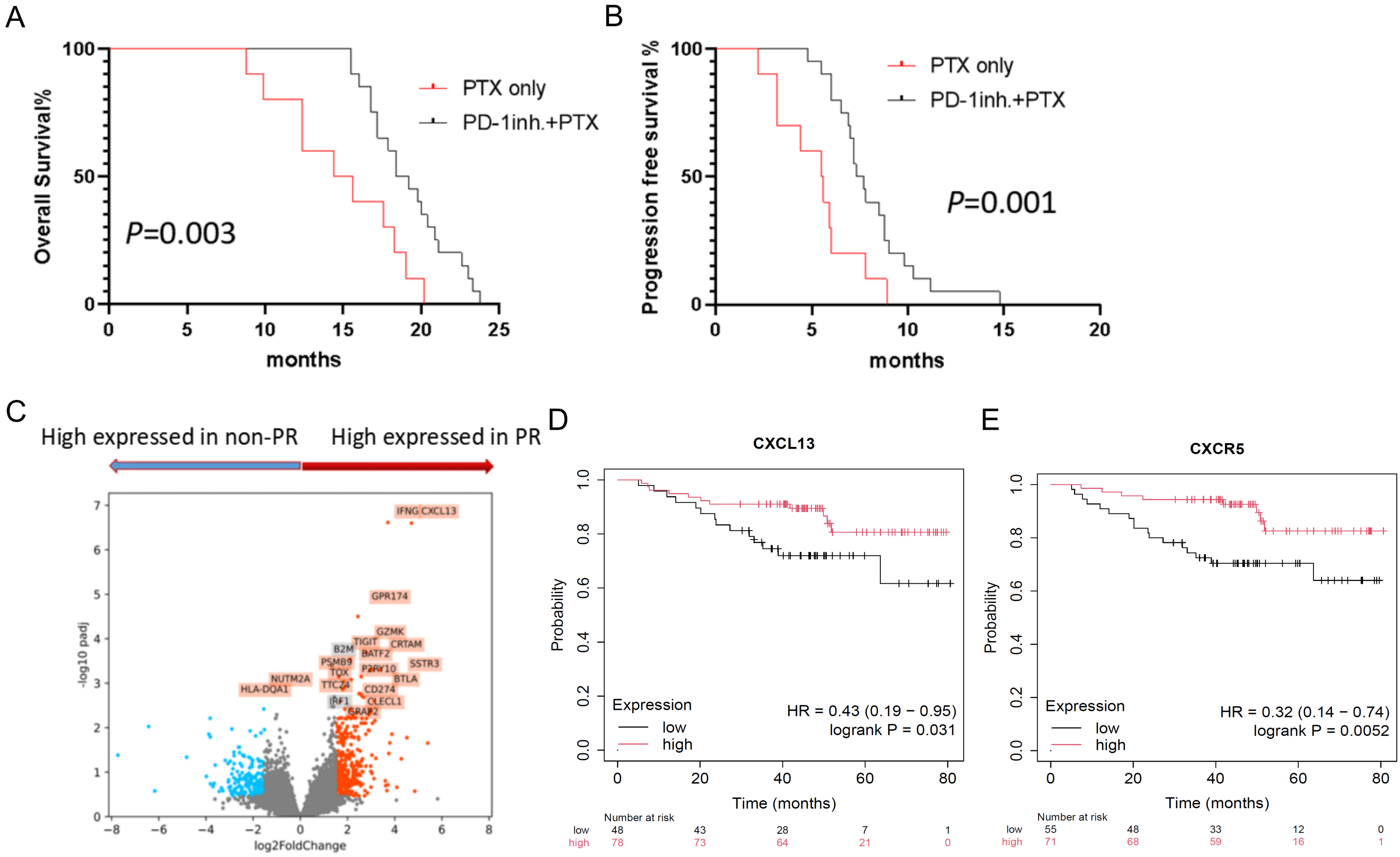

Supplemental Figure S1 Combination of paclitaxel and Tislelizumab improves survival in patients with triple-negative breast cancer (TNBC). (A-B) Overall survival (OS) and progression-free survival (PFS) curves for TNBC patients under PTX and Tis treatment. n =10 per/cohort. Kaplan-Meier log-rank test. **(C)** Baseline CXCL13 expression levels in patients comparing the pCR (pathological complete response) group with the non-pCR group in the prospective single-arm phase II cohort evaluating paclitaxel combined with Tislelizumab for TNBC under RNAseq analysis. **(D, E)** KM plotter analysis of the correlation between CXCL13/CXCR5 gene expression and prognosis (OS) in TCGA-TNBC patients. Median value was used as the cutoff criteria.

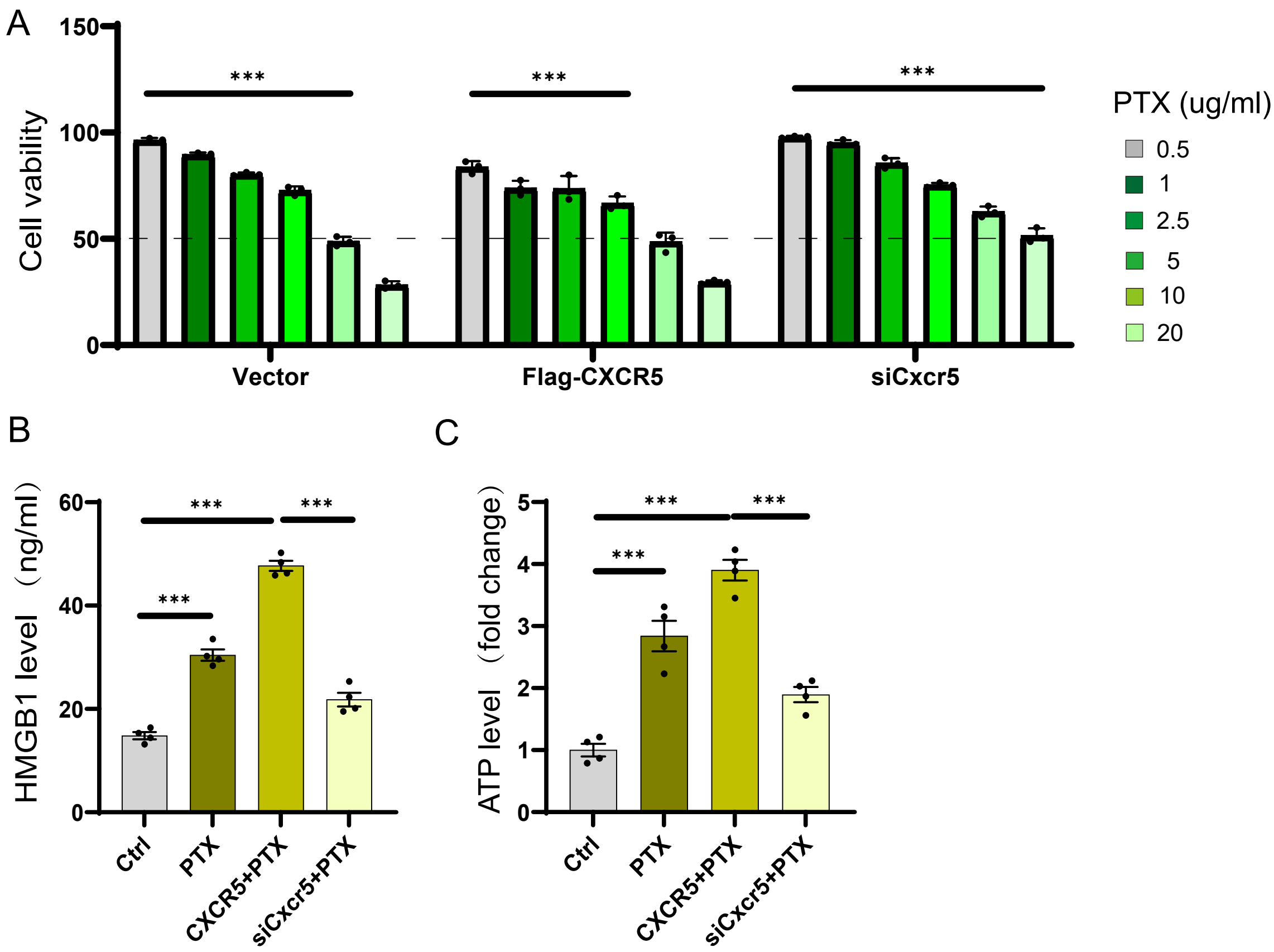

Supplemental Figure S2 CXCR5 knockdown reduces cell viability, HMGB1 release, and ATP levels in MDA-MB-231 cells treated with paclitaxel (PTX) and Tislelizumab (Tis). (A) Cell viability of MDA-MB-231 cells transfected with Flag-CXCR5, CXCR5 siRNA (siCxcr5) or control vector (Vector) after PTX treatment at indicated concentrations (0, 0.5, 1, 2.5, 5, 10, 20 μg/ml). **(B)** HMGB1 levels in culture supernatants of MDA-MB-231 cells under control (Ctrl), PTX, CXCR5 overexpression (CXCR5+PTX), or siCxcr5+PTX conditions. **(C)** ATP levels (fold change) in MDA-MB-231 cells under the same treatment conditions. Data are presented as mean ± SEM, ***p < 0.001 (one-way ANOVA with Tukey ’ s multiple comparisons test).

A

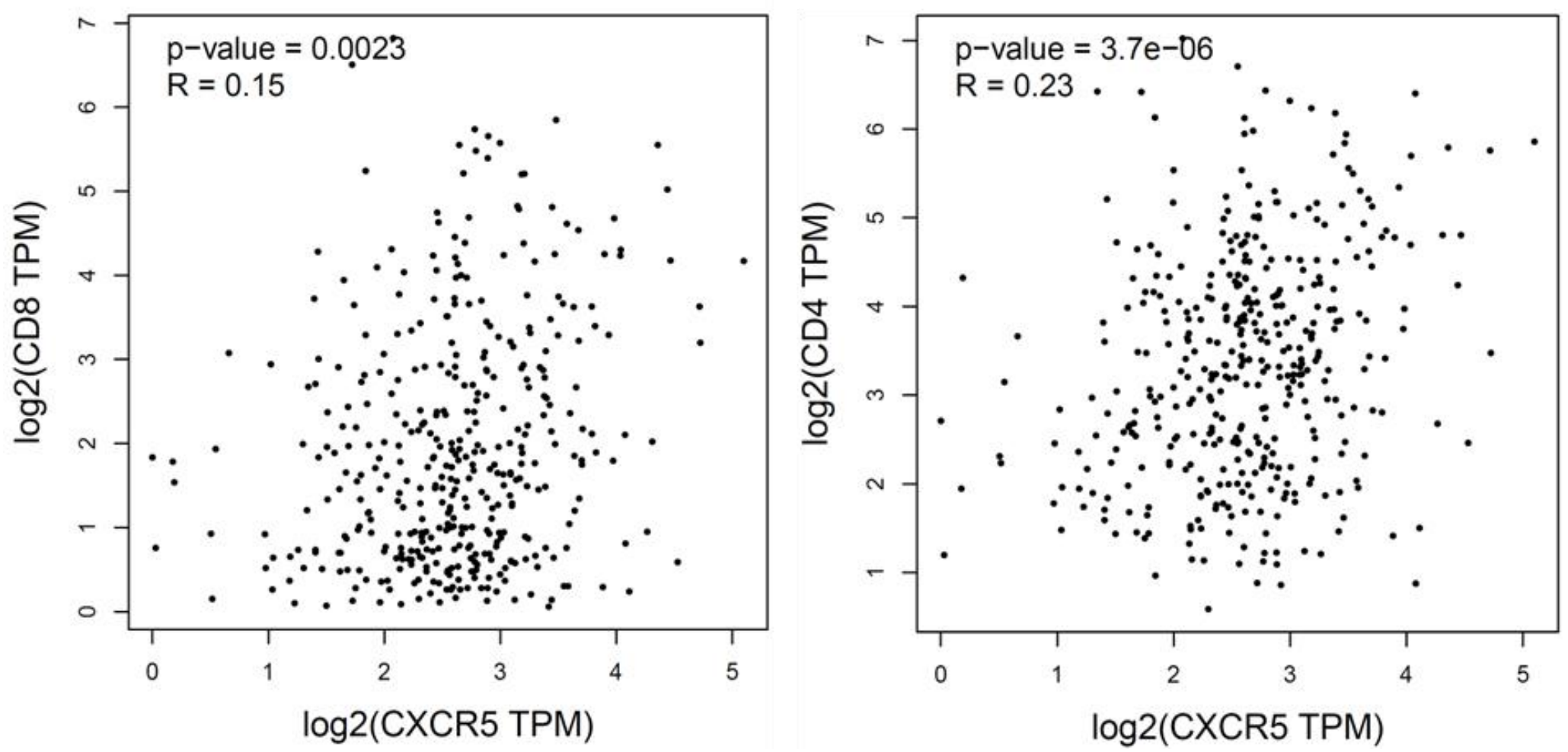

B

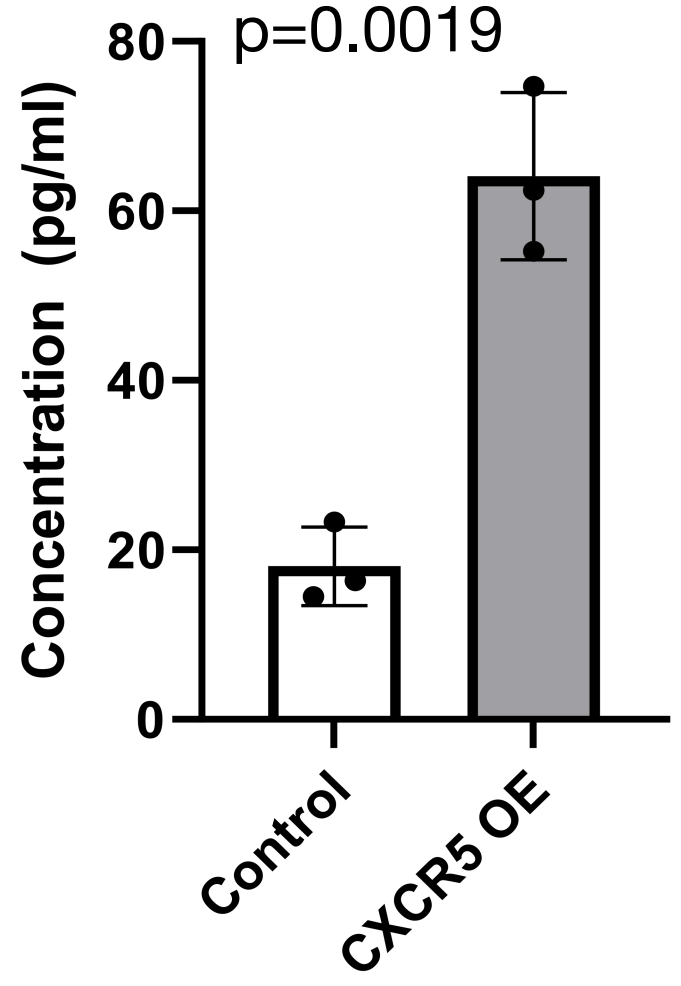

Supplemental Figure S3 Database analysis of the association between CXCR5 and CD4+T,CD8+T cells infiltration and CXCL13 expression. A. CXCR5 expression levels are significantly and positively correlated with CD4 and CD8 T-cell infiltration from using large-scale public GEPIA datasets. **B.** CXCR5 overexpression associates with an increased CXCL13 level (P=0.0019)(*t* text).

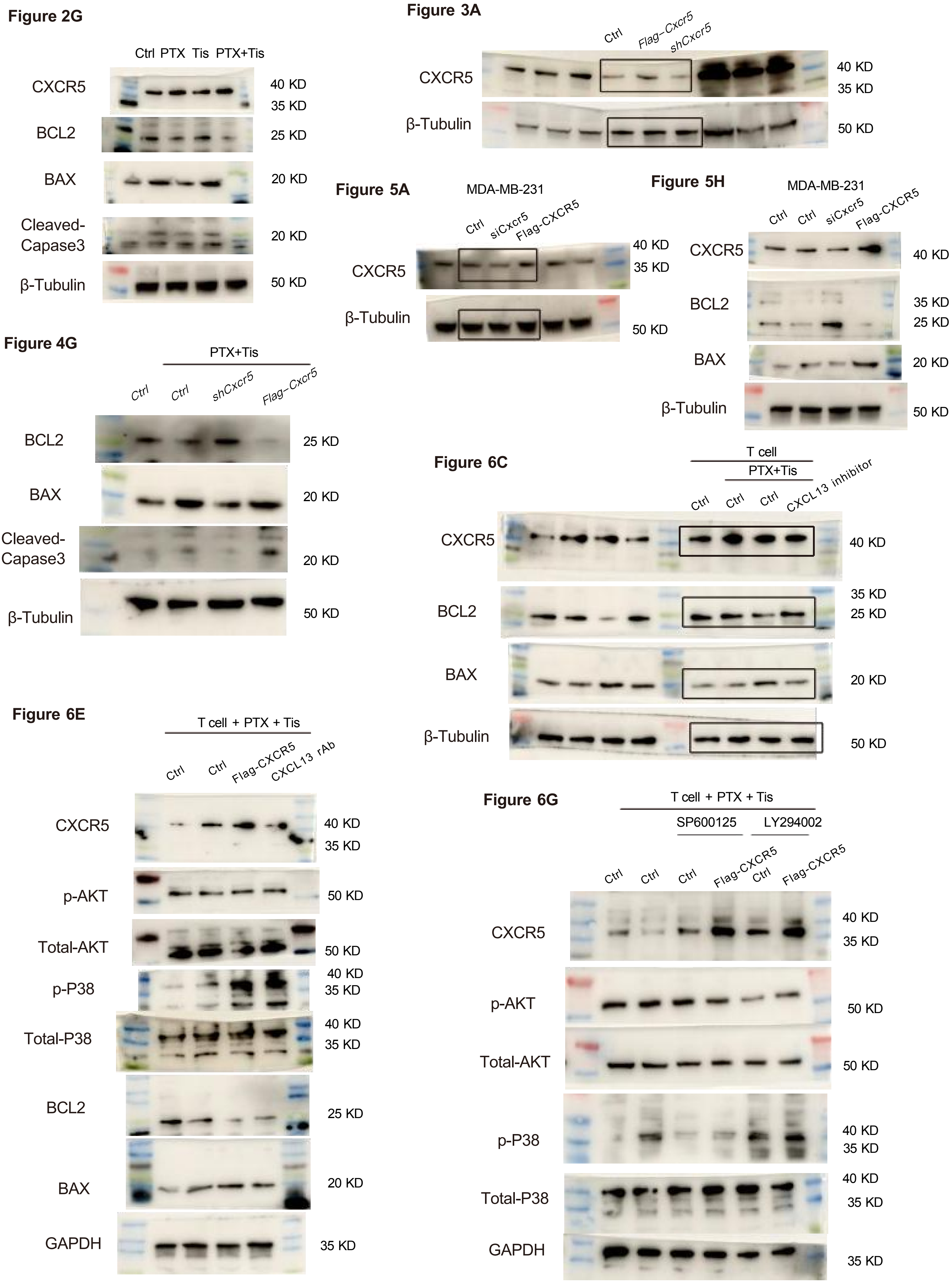

Supplemental Figure S4 Original images of all Western-blot assays of the study. Uncropped full original blots for all major blots presented in main Figures 2-6.
